# Feasibility and Acceptability of Global Positioning System (GPS) Methods to Study the Spatial Contexts of Substance Use and Sexual Risk Behaviors among Young Men Who Have Sex with Men in New York City: A P18 Cohort Sub-Study

**DOI:** 10.1371/journal.pone.0147520

**Published:** 2016-02-26

**Authors:** Dustin T. Duncan, Farzana Kapadia, Seann D. Regan, William C. Goedel, Michael D. Levy, Staci C. Barton, Samuel R. Friedman, Perry N. Halkitis

**Affiliations:** 1 Department of Population Health, New York University School of Medicine, New York, NY, United States of America; 2 College of Global Public Health, New York University, New York, NY, United States of America; 3 Population Center, New York University, New York, NY, United States of America; 4 Center for Health, Identity, Behavior and Prevention Studies, New York University, New York, NY, United States of America; 5 Center for Drug Use and HIV Research, New York University College of Nursing, New York, NY, United States of America; 6 Center for Data Science, New York University, New York, NY, United States of America; 7 Institute of Infectious Disease Research, National Development and Research Institutes, Inc., New York, NY, United States of America; Asociacion Civil Impacta Salud y Educacion, PERU

## Abstract

**Background:**

No global positioning system (GPS) technology study has been conducted among a sample of young gay, bisexual, and other men who have sex with men (YMSM). As such, the purpose of this study was to evaluate the feasibility and acceptability of using GPS methods to understand the spatial context of substance use and sexual risk behaviors among a sample of YMSM in New York City, a high-risk population.

**Methods:**

Data came from a subsample of the ongoing P18 Cohort Study (*n* = 75). GPS feasibility and acceptability among participants was measured with: 1) a pre- and post-survey and 2) adherence to the GPS protocol which included returning the GPS device, self-report of charging and carrying the GPS device as well as objective data analyzed from the GPS devices. Analyses of the feasibility surveys were treated as repeated measures as each participant had a pre- and post-feasibility survey. When comparing the similar GPS survey items asked at baseline and at follow-up, we present percentages and associated p-values based on chi-square statistics.

**Results:**

Participants reported high ratings of pre-GPS acceptability, ease of use, and low levels of wear-related concerns in addition to few concerns related to safety, loss, or appearance, which were maintained after baseline GPS feasibility data collection. The GPS return rate was 100%. Most participants charged and carried the GPS device on most days. Of the total of 75 participants with GPS data, 75 (100%) have at least one hour of GPS data for one day and 63 (84%) had at least one hour on all 7 days.

**Conclusions:**

Results from this pilot study demonstrate that utilizing GPS methods among YMSM is feasible and acceptable. GPS devices may be used in spatial epidemiology research in YMSM populations to understand place-based determinants of health such as substance use and sexual risk behaviors.

## Introduction

Spatial epidemiology has made important contributions in understanding connections between physical and social neighborhood environments and health and health behavior [1]. The existing research among men who have sex with men (MSM) shows neighborhood factors can influence their drug use and high-risk sexual behaviors [2–9]. For example, one study in 385 young MSM (YMSM) in New York City found that neighborhood-level gay presence was significantly and positively associated with consistent condom use during anal intercourse [3]. Consistent with several other studies on neighborhoods and MSM health [2–9], a limitation of this study was that the neighborhood unit of analysis used was the residential ZIP code. This is problematic as the field of spatial epidemiology is fraught with several issues including spatial misclassification, where a neighborhood-level exposure is characterized incorrectly [10], and the “residential trap,” where static geographic information systems (GIS) approaches limit the study of exposure to the residential area [11, 12]. These methodological issues are important and inhibit our understanding of neighborhood effects on health, e.g., simulation research suggests that defining neighborhoods with too large a spatial unit (e.g. an administrative unit) will underestimate the neighborhood effect on a health outcome [13, 14]. GIS methods usually employ administratively defined neighborhood boundaries (i.e. census tracts or ZIP codes) or spatial buffers around a geocoded location known as static egocentric buffers without recognizing that the contexts in which individuals spend time are not limited to these defined areas [10]. Furthermore, standard GIS techniques do not capture individual travel patterns and behaviors, and GIS methods make isotropic assumptions regarding one’s exposure to specific neighborhood environments, i.e. assumes equal movement in all directions. This issue is known as the uncertain geographic context problem, which arises because of the spatial uncertainty in the actual neighborhoods that exert contextual influences on the individuals being studied and the temporal uncertainty in the timing and duration in which individuals experienced these contextual influences [15]. The neighborhood research in MSM populations is similar to the vast majority of general neighborhoods and health research, whereby most neighborhood health research among MSM suffers from the aforementioned limitations of GIS-based research and focus exclusively on their residential neighborhoods. The concept of “spatial polygamy”, however, argues that people experience and interact with multiple neighborhoods, which can influence their health [16]. Like other populations, MSM are spatially polygamous, including in urban areas such as New York City [17, 18]. For example, one recent study assessing concordance between neighborhoods (defined as the five boroughs of New York City) in which YMSM reside, socialize, and have sex in found that 75% of these men reported discordance between all three of these borough types [17].

Studies are increasingly using mobility data to assess the impact of multiple contextual exposures. There are different approaches to assessing spatial mobility and spatial polygamy including the use of travel diary, web-based assessment of regular destinations as well as use of Global Positioning System (GPS) data. For example, VERITAS (Visualization and Evaluation of Route Itineraries, Travel Destinations, and Activity Spaces) is a web-based application nested within a computer-assisted personal interview that allows researchers to assess daily mobility, travel to regular destinations, and perceived neighborhood boundaries using interactive mapping technology [19] and has been used in previous spatial epidemiology research [20, 21]. Each method to examine spatial mobility has strengths and limitations; for example, a limitation of surveying a participant’s regular locations can be recall bias and this method can be implicated in spatial misclassification. A recent web-based mapping study in MSM found locational errors when participants identified locations, including when they mapped their homes (the median home location error across participants was 0.65 miles) [22]. A well-suited alternative method to address these issues is the use of GPS technology [23, 24], which can record mobility patterns in real-time to assess lengths of exposure to various spatial contexts. While we are not aware of any GPS studies done in MSM populations, there has been some GPS feasibility research conducted among certain marginalized groups such as drug-using populations [25–28]. For example, Mirzazadeh and colleagues [27] conducted focus group discussions among injecting drug users and found that participants identified several concerns about carrying GPS devices, including what authorities might do with the data, that other people who inject drug and dealers may suspect them as informants, and adherence to carrying and use. However, most participants felt concerns were surmountable with detailed informed consent on the purpose of the study and practical ways to carry, charge, and hide devices [27]. Results from a study that conducted focus group discussions among adults in Iquitos, Peru found concerns regarding whether the GPS unit would audio/videotape them and concerns regarding the confidentiality of information [29]. This suggests the need for research assessing the feasibility and acceptability of GPS methods to studying neighborhood contexts among MSM, who may mistrust medical research and research more broadly [30, 31] and because past feasibility of GPS research might not be applicable across population groups.

We focused on YMSM in this study because they are a high-risk group, especially in urban areas such as New York City. As an example, MSM comprise the largest group of individuals in the United States (U.S.) infected with HIV [32]. Stark disparities in HIV prevalence and incidence continue to persist among MSM, especially YMSM [33]. In 2010, YMSM (aged 13–24) accounted for 72% of all new HIV infections among all persons aged 13–24 years, and nearly one-third of new HIV infections among all MSM, with a 22% increase in the number of new HIV infections in this group from 2008–2010 [34]. Rates of new HIV infections are greatest in metropolitan areas with populations of 500,000 people compared to smaller metropolitan and non-metropolitan areas [34]; thus, HIV disparities are greatest in urban areas such as New York City [35], so New York City is an ideal location to conduct this research. Additionally, substance use is highly prevalent among YMSM. A recent nationally representative study found YMSM, who were between the ages of 15 and 24, were more likely to have used marijuana or powder cocaine in the past year and more likely to have engaged in binge drinking in the past year than their heterosexual counterparts [36]. Our prior research with the Project 18 (P18) cohort study demonstrates that past month substance use (e.g. alcohol use and marijuana use) as well as past month condomless sexual behavior (e.g. unprotected receptive oral intercourse and unprotected receptive anal intercourse), as measured using a Timeline Follow Back method [37], increased between baseline and 18-month follow-up [38]. The purpose of this novel study is to evaluate the feasibility and acceptability of utilizing GPS technology in a sample of YMSM in New York City in the P18 cohort study (regardless of their drug use, sexual risk behaviors and HIV status)—employing a state-of-the-science GPS technology.

## Materials and Methods

### The P18 Cohort Study

Participants in this study were enrolled in Project 18 (P18), a prospective cohort study of sexual behavior, substance use and mental health burdens including 600 racially/ethnically and socioeconomically diverse YMSM recruited between June 2009 and May 2011. The P18 cohort study has been described in detail elsewhere [39, 40]. Briefly, participants were eligible for this study if they were 18 or 19 years old, biologically male, lived in the New York City metropolitan area, reported having had sex (any physical contact that could lead to orgasm) with another male in the 6 months preceding screening, and self-reported a HIV-negative or unknown serostatus. P18 participants predominantly lived in one of the New York City boroughs (69%) [17]. For this GPS sub-study, our goal was to recruit 25 Black men, 25 Hispanic men and 25 White men to participate in this study. To achieve this sample, we emailed n = 200 active study participants of which n = 82 individuals called in to be screened, n = 81 were screened, and n = 75 were scheduled to come in to obtain their GPS devices and take a brief GPS feasibility survey and a survey on perceived neighborhood characteristics (e.g., exposure to neighborhood crime, neighborhood social norms about drug use and sex/HIV and gay friendliness of the neighborhood). Participants were compensated $25 for their first 30 minute visit and $75 at their second visit after returning a week later with the GPS device and charging materials. Assessments for the GPS sub-study were conducted between October and November 2014 at New York University’s Center for Health, Identity, Behavior and Prevention Studies (CHIBPS). Other survey data including substance use, sexual behavior, HIV status and socio-demographics were assessed during a prior office visit and were conducted beginning in July 2014. The Institutional Review Board at New York University approved the research protocol and written informed consent was obtained prior to participation in this study.

### GPS Protocol and Data Processing

In this study, we used QStarz’s BT-Q1000XT GPS Travel Recorder (Qstarz International Co., Ltd., Taipei, Taiwan), which has been used in multiple prior research studies [41–50]. Also consistent with other research studies [42, 44, 47, 50–56], GPS tracking of the sample was conducted for a week, i.e. seven (7) days and participants in this study generally wore the GPS device 5 week days and 2 weekend days. We followed a GPS protocol used in past work where the GPS device was set to record locations in 30-second intervals [23]. Prior to implementing the GPS study protocol, key study staff (e.g. Project Directors) participated in the GPS Overview and Training Workshop. The purpose of the workshop was to provide an overview of GPS technology and to review the GPS protocol so that the protocol was implemented with integrity. In the workshop, staff members were trained to use the GPS units and were taught important points to discuss with participants (e.g. how to wear/carry the GPS unit). Participants were instructed to place the small GPS device in their pockets or bags, if their clothing did not have any pockets, and to keep the device with them during the study orientation and baseline assessment. Participants also were instructed to charge the GPS devices nightly and to wear the GPS devices at all times (except when sleeping, swimming or showering). We additionally asked participants to complete a travel diary, which consisted of questions that asks: “Did you charge the GPS monitor today?” (Response options: “Yes” and “No”) and “Did you carry the GPS monitor with you today?” (Response options: “Yes—for all journeys”, “Yes—for some journeys”, “No—but did not make journeys” and “Did not travel today”)–which was meant to help the participant remember to charge the unit and carry it with him throughout the week. The GPS device (battery fully charged and with a unique study GPS serial number) was given to participants in a large zip lock bag, which also contained a mini USB charging cord for the GPS device, a USB wall adapter for charging, a pamphlet containing background information on GPS technology, and a travel diary. Participants were reminded daily (except Sunday) via text to charge their GPS devices. Upon completion of our weeklong GPS protocol (i.e. carrying the device for all journeys, charging the device daily, and completing the travel diary), participants came back to our research office to return the GPS devices at which time participants completed the GPS feasibility and acceptability survey and were given the larger incentive. Whenever a participant forgot to fill out their travel diary (which was relatively rare), project staff members filled the diary out jointly with participants during this follow-up visit.

Several security measures were put in place to protect the GPS mobility data of our participants. First, as previously described, all GPS devices had a unique ID number that was separate from the participant ID number. Second, the GPS data was downloaded by project staff on a single computer in our office that was password protected and in a locked office. The geospatial analyst was sent the GPS data via a secure password protected web-based system and was not given attribute data of any participant. Strict data confidentiality was maintained in this study. GPS data was downloaded using the Qstarz proprietary software and stored as.gpx files. The.gpx files, were transformed into shapefiles, and stored in an ESRI geodatabase for further analysis and map creation. GPS data were processed with several scripts, and with models built using ArcGIS version 10.2 (ESRI, Redlands, CA) and the Python programming language (Python Software Foundation. Python Language Reference, version 2.7. Available at http://www.python.org).

### GPS Feasibility and Acceptability

GPS feasibility and acceptability was measured in multiple ways. First, we measured GPS feasibility and acceptability using survey-based methods, consistent with previous research [23, 51, 57, 58]. In particular, we implemented a pre- and post-survey including 7 items that were similar across both surveys to facilitate comparisons [23, 57]. For example, at baseline participants were asked “GPS makes it more interesting to participate in the study” and “I am worried about someone trying to steal the GPS,” while at follow up, participants were asked “GPS made it more interesting to participate in the study” and “I was worried about someone trying to steal the GPS” [23, 57]. Participants were asked to use a Likert scale from 1 (strongly agree) to 5 (strongly disagree) for these items [23, 57]. Similar to past research [23], the survey also included 21 post-only GPS questions, including “Overall, was it easy to use the GPS?”; “Did you feel comfortable wearing the GPS?”; “I would participate in another GPS research study”; and “Would you participate in a GPS study that tracked you for two weeks?” Response options for the majority of these questions were “Yes” and “No”.

Second, we evaluated adherence to the GPS protocol, which included returning the GPS device, self-report of charging and carrying the GPS device (as assessed via the travel diary), and objective data analyzed from the GPS devices (i.e., the total number of GPS data fixes per participant, the number of days the devices were worn per participant, and the amount of GPS data obtained from the GPS device per day) [23, 46, 58–60]. GPS data may be analyzed in various ways depending upon the desired unit of analysis; here we are interested in activity spaces. We did not require that these points necessarily be consecutive in nature because we were assessing overall wear time. In this study, we examined the number of days of wear time of the GPS unit per participant and used various cutoff values to determine GPS wear time across the study participants. Valid and useable GPS data for a day was defined if a participant had at least 120 GPS data points in a given day as this would indicate an hour of wear time with our GPS device attempting to log in 30 second intervals, which is a threshold used in previous GPS research [23]. As additional measures applied in previous GPS research, we used a cutoff of 5 hours, 8 hours and 12 hours of wear time to ascertain GPS data quality [23, 51]. Finally, we present a map of a participants’ GPS data as an additional measure of feasibility. There are different ways to map GPS data [61]. We recognize that caution should be taken when mapping GPS data, including when mapping YMSM, due to concerns regarding confidentiality. However, in this case, New York City is the most populous city and most densely population metropolitan area in the U.S., including a large YMSM population, so we believe that there is little risk of identification given that all other demographic information is presented in aggregate. We have also chosen to obscure the participants GPS data occurring while at their home neighborhood (ZIP code). That is, GPS points in the participant’s ZIP code were hidden to protect confidentiality.

### Socio-Demographic Characteristics

We collected data on sociodemographic characteristics such as: age (years), gender (male, transfemale, genderqueer), ethnicity (Hispanic or non-Hispanic) and race (Black, Asian-Pacific Islander, Native American, White, multiracial and other). Participants reported whether or not they were currently enrolled in school (yes, no) as well as their highest level of education completed (high school or less, some college/ technical school, college degree or more). Participants were also asked where they currently lived: in a family apartment/house, their own apartment/house, with friends/roommates or in temporary housing/shelter. They reported their total individual annual income which is categorized here as <$15,000, $15,000-$35,000, >$35,000 which approximates national poverty level (<$15,000) and above (>$15,000). Data were also collected on sources of income including: regular job, public assistance, odd jobs, own business, parent, selling drugs and sex for money/drugs. We assessed sexual identity using the 6-point Kinsey scale, which ranges from 0-exclusively heterosexual to 6-exclusively homosexual. For the purpose of this analysis, sexual identity was dichotomized as exclusively homosexual versus not exclusively homosexual. To assess for relationship status, participants reported whether or not they currently had a main/stead partner (yes, no). In addition, participants reported if they were foreign born (yes, no). Participants were also asked to report their HIV status. If they reported being negative, unknown or positive without proof of status, their status was confirmed with the INSTI^™^ HIV-1/HIV-2 Rapid Antibody Test from bioLytical^™^ Laboratories. All positive test results were further verified with a confirmatory HIV test.

### Statistical Analysis

First, we computed descriptive statistics on the socio-demographic data. After this, we examined the feasibility of GPS methods among the sample by computing descriptive statistics on the GPS feasibility and acceptability measures. IBM SPSS statistics software (version 21.0; IBM Corp, Armonk, NY) was used to conduct the statistical analyses. The analytic sample for the survey data included participants who answered the pre (*n* = 75) and post feasibility surveys (*n* = 74). Analyses of the feasibility surveys were treated as repeated measures as each participant had a pre- and post-feasibility survey. When comparing the similar GPS survey items asked at baseline and at follow-up, we present percentages and associated *p*-values based on chi-square statistics computed from McNemar's test for related samples [62]. Statistical significance was determined at *p* < 0.05. The GPS map of a participant was created in ArcGIS (ESRI, Redlands, CA).

## Results

### Socio-Demographics

Table 1 shows the sample socio-demographics of the YMSM. Most participants were 22 or 23 years of age (92%) and identified as male (93%). By design, most participants were Black, Hispanic, or White (96%). Most were currently not enrolled in school (68.0%) and lived with their family (41.3%). The annual individual-level income was <$15,000 for approximately 50% of the participants, with the main source of income being a regular job. The vast majority (87%) of participants were born in the U.S. Almost half (46%) of the sample completed high school or less education. Almost two-thirds (60%) reported being exclusively homosexual and about 47% reported currently having a partner. A very small percent of the sample was HIV-positive (4%).

**Table 1:** Sample Socio-Demographics (*n* = 75).

	% (*n*)
**Age**	
21	8.0 (6)
22	45.3 (34)
23	46.7 (35)
**Gender**	
Male	93.3 (70)
Transfemale	1.3 (1)
Genderqueer	2.7 (2)
No gender identification	2.7 (2)
**Race/Ethnicity**	
Black	33.3 (25)
Hispanic/Latino	37.3 (28)
White	25.3 (19)
Asian-Pacific Islander	6.7 (5)
Other (e.g. Mixed)	6.7 (5)
**Currently enrolled in school** (yes)	32.0 (24)
**Education**	61.3 (46)
High school or less	10.7 (8)
Some college / technical school	28.0 (21)
College degree or more	
**Current Housing**	
Family apt/house	41.3 (31)
Own apt /house	18.7 (14)
Friends/roommates	28.0 (21)
Temporary housing/Shelter	11.9 (9)
**Annual Income (total, individual)**	
<$15,000	49.3 (37)
$15,000-$35,000	37.3 (28)
>$35,000	10.6 (8)
DK/RTA	2.6 (2)
**Sources of Income**	
Regular Job	81.3 (61)
Public Assistance	20.0 (15)
Odd jobs	40.0 (30)
Own business	12.0 (9)
Parent	69.3 (52)
Selling drugs	5.3 (4)
Sex for money/drugs	6.7 (5)
**Foreign-born** (yes)	13.3 (10)
**Sexual identity** (exclusively homosexual)	60.0 (45)
**Currently has a partner** (yes)	47.3 (35)
**Confirmed HIV positive**	4.0 (3)

### GPS Feasibility and Acceptability

Overall, we found that it was feasible and acceptable to use GPS devices among our sample of YMSM. Seventy-five YMSM agreed to participate in the study where one of the criteria was wearing a GPS device for a week and all GPS devices were returned (100%).

Table 2 compares GPS feasibility pre- and post-survey measures. For both pre- and post-survey measures, participants reported high ratings of GPS acceptability and ease of use and low levels of wear-related concerns in addition to few concerns related to safety, loss, or appearance. Therefore, GPS feasibility was maintained after baseline GPS feasibility data collection. These results remained stable from pre to post assessment with the exception of two survey measures. At baseline and follow-up participants were asked "The GPS seems uncomfortable to wear" and "The GPS irritated my skin or was uncomfortable to wear", respectively, where 6.7% and 0% (*p* = .063) reported they "strongly agree or agree". This marginally significant result suggests that participants became more comfortable with the GPS device once they had used it. Furthermore, at baseline and follow-up participants were asked, “I am concerned that I will lose the GPS" and "I was concerned that I would lose the GPS", respectively, where 8% and 17.6% (*p* = .039) reported that they "strongly agree or agree". This result suggests that participants became more concerned about losing their GPS device after participating in the GPS protocol. However, it is worth noting that though there was a significant increase in concern over losing the GPS, the overall concern with losing the GPS at post-GPS protocol assessment was still relatively low (17.6%) and no GPS devices were lost over the course of the weeklong protocol. Finally, at baseline and follow-up participants were asked, “I am concerned about how I will look wearing the GPS” and “I was concerned about how I looked wearing the GPS”, respectively, where 10.7% and 2.7% (*p* = 0.267) reported that they "strongly agree or agree".

**Table 2:** Comparison of Pre- and Post-GPS Feasibility and Acceptability Survey Items.

Pre-GPS survey (*n* = 75)		Post-GPS survey (*n* = 74)		
Question*	% Strongly Agree/Agree*	Question*	% Strongly Agree/Agree*	*p*-value**
1. I am comfortable with the research study tracking where I go using GPS.	93.3	1. I felt comfortable with the research study tracking where I go using GPS.	89.2	0.375
2. GPS makes it more interesting to participate in the study.	81.3	2. GPS made it more interesting to participate in the study.	82.4	0.999
3. I am worried about someone trying to steal the GPS.	6.7	3. I was worried about someone trying to steal the GPS.	4.1	0.625
4. The GPS seems uncomfortable to wear.	6.7	4. The GPS irritated my skin or was uncomfortable to wear.	0	0.063
5. I am concerned that I will lose the GPS.	8.0	5. I was concerned that I would lose the GPS	17.6	0.039
6. I am worried about my safety wearing the GPS.	2.7	6. I worried about my safety wearing the GPS.	0	0.500
7. I am concerned about how I will look wearing the GPS.	10.7	7. I was concerned about how I looked wearing the GPS.	2.7	0.267

* Other response options for questions were “neutral” and “strongly disagree/disagree” (data not shown).

** *p*-values are based on chi-square statistics, comparing pre-GPS and post-GPS survey questions. McNemar’s test (McNemar, 1947) was used to account for the repeated measures design.

The GPS feasibility post survey items overall also demonstrate that use of GPS was viewed as feasible by participants (Table 3). For example, when asked “Overall, was it easy to use the GPS?” 97.3% reported “yes.” Approximately 93% of participants reported “yes” to “Did you feel comfortable wearing the GPS?” To the question, “Did the GPS device get in the way of your everyday activities?” 97% reported “no.” In addition, 98.6% of the sample answered, “yes” to “I would participate in another GPS research study” and 100% answered “yes” to “Would you participate in a GPS study that tracked you for two weeks?”

**Table 3:** GPS Feasibility and Acceptability Post Survey Items (*n* = 74).

	Response (%)	
Questions	No	Yes
1. I had issues or problems with the GPS device during the study.*	94.6	4.1
2. Did you have problems turning the GPS device on or off?	98.6	1.4
3. Did you forget to charge the GPS device at night?	73.0	27.0
4. Did you forget where to put the GPS device?	100	0
5. Do you think the GPS device was too big?	79.9	20.3
6. Do you think the GPS device was too small?	98.6	1.4
7. Did the GPS run out of battery during the day?	75.3	24.7
8. Overall, was it easy to use the GPS?	2.7	97.3
9. Did you have any problems carrying or wearing the GPS?	95.5	4.1
10. Were you able to solve any problems you had with the GPS?	18.6	81.4
11. Did you feel comfortable wearing the GPS?	6.8	93.2
12. Did the GPS device get in the way of your everyday activities?	97.3	2.7
13. Was the battery life of the GPS too short?	82.4	17.6
14. Did you forget to wear the GPS device daily?	89.2	10.8
15. I would participate in another GPS research study.	1.4	98.6
16. Did using the GPS device cause you to alter your behavior?	98.6	1.4
17. Were there any activities that were difficult to do with the GPS on?	91.9	8.1
18. Was the GPS device inconvenient to carry/wear?	90.5	9.5
19. Was it a chore to wear the GPS device?	82.4	17.6
20. Did you like the look of the GPS device?	50	50
21. Would you participate in a GPS study that tracked you for two weeks?	0	100

* For question #1 (“I had issues or problems with the GPS device during the study”), response options were as follows “strongly agree”, “agree”, “don’t know”, “strongly disagree” and “disagree” where “Yes” is “strongly agree/agree” and “No” is “strongly disagree/disagree”.

Table 4 shows an aspect of the GPS protocol compliance, self-reported carrying and charging of the GPS unit, based on the travel diary. We found that, for the most part, participants charged and carried the GPS device on most days. For example, the percentage of participants that charged their GPS for the day ranged from 97.3% to 88.0% for all 7 days of the study. The percentage of participants that carried their GPS on at least some of their daily travels ranged from 96.0% to 82.7% for all 7 days of the study.

**Table 4:** Travel Diary Reported GPS Charging and Carrying (*n* = 75).

***“Did you charge the GPS monitor today*?*”***				
	**Yes (%)**	**No (%)**		
**Day**				
1	69 (92)	6 (8)		
2	71 (94.7)	4 (5.3)		
3	73 (97.3)	2 (2.7)		
4	68 (90.7)	7 (9.3)		
5	66 (88)	8 (10.7)		
6	67 (89.3)	6 (8)		
7	66 (88)	8 (10.7)		
***“Did you carry the GPS monitor with you today*?*”***				
	**Yes—for all journeys (%)**	**Yes—for some journeys (%)**	**No—but did make journeys (%)**	**Did not travel today (%)**
**Day**				
1	68 (90.7)	4 (5.3)	0 (0)	2 (2.7)
2	61 (81.3)	6 (8)	0 (0)	8 (10.7)
3	59 (78.7)	3 (4)	2 (2.7)	11 (14.7)
4	56 (74.7)	10 (13.3)	0 (0)	9 (12)
5	59 (78.7)	8 (10.7)	0 (0)	8 (10.7)
6	63 (84)	4 (5.3)	4 (5.3)	4 (5.3)
7	68 (90.7)	3 (4)	1 (1.3)	3 (4)

Objective GPS data from all participants over all days resulted in a total N of 958,716 GPS data records written into.gpx files. There were likely data errors due to multipath reflectance and timing issues; in addition there were some duplicate data. Multipath reflectance is the process by which signals between GPS satellites and GPS receiver “bounce” or reflect off tall buildings; when the signal connects with the GPS receiver this reflectance causes errors in the location calculation. Here we were looking at activity spaces and areas visited, so we were not concerned with small-scale (i.e. 10–20 meter) variation. Regardless, in an attempt to minimize the effect of data errors on the analysis we applied several techniques to minimize these effects. First, duplicate.gpx data were removed from analysis. Second, data that had either erroneous timestamps (i.e. year 1980) or data with erroneous Latitude or Longitude values (0, 0) were removed. Finally, a nearest neighbor analysis with a distance threshold of 100m was run on data files from each participant in QGIS to remove GPS data points with sudden large jumps in position. These techniques remove a minimal amount of data (N = 5,024 <0.005%), leaving a total sample size of 953,692. The total number of GPS points per participant for the duration of the study varied from a low of 1,739 points (representing approximately 14.5 hours of data) to a high of 22,135 points (representing approximately 184.5 hours of data) and mean count of 12,715 per participant (representing approximately 106.0 hours of data). Of the total of 75 participants with GPS data, all 75 (100%) had at least one hour of GPS data for one day as indicated by 120 GPS fixes on a day given our 30-second sampling rate and 63 (84.4%) had at least one hour (120 GPS points) on 7 or more days (Table 5). When participant data (n = 75) was disaggregated by the number of hours participants wore the GPS, we found that participants had a range of viable data. Using a cutoff of 12 hours of wear time indicating a full day of GPS use (indicated by 1440 GPS data points on any given day), 28 of 75 participants or 37.3% had at least 12 hours of wear time on 7 or more days.

**Table 5:** Number of days with GPS data per participant, by different time thresholds (*n* = 75).

	GPS Time Threshold			
Days*	1-hour (Percent)	5-hour (Percent)	8-hour (Percent)	12-hour (Percent)
1	100	100	96.0	90.7
2	100	94.7	90.7	86.7
3	100	93.3	86.7	82.7
4	98.7	88	80.0	69.3
5	93.3	84	72.0	62.7
6	92.0	73.3	66.7	53.3
> = 7	84.0	58.7	50.7	37.3

* These days are not necessarily consecutive but rather meet our threshold.

**Fig 1:**
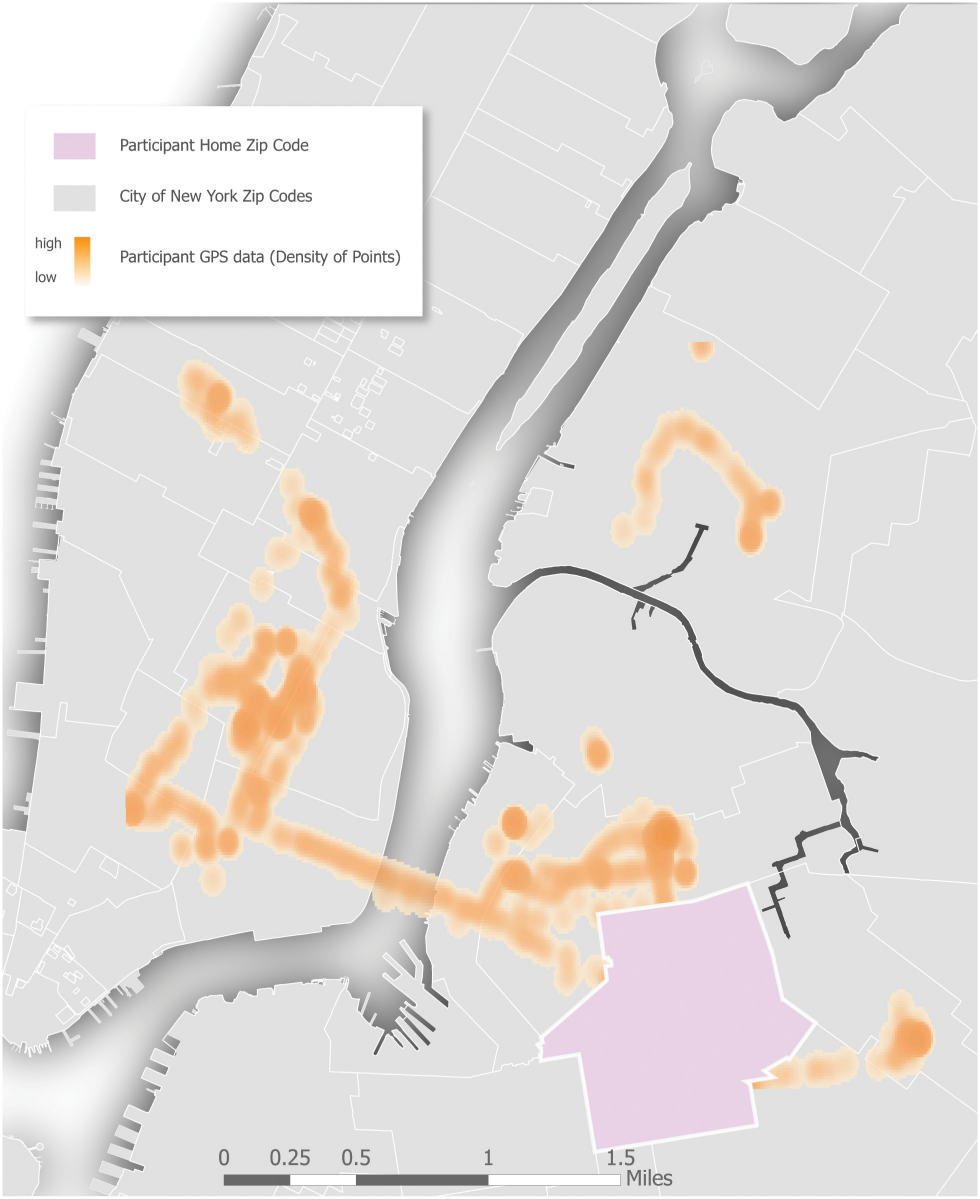
GPS tracks from a P18 GPS Sub-study participant. Note: “High” means a high density of GPS points, whereas “low” means a low density of GPS points. GPS points located in participants’ home ZIP code were hidden to protect participant privacy.

Fig 1 is a map of the raw GPS data, from one participant, overlaid onto their residential ZIP code. This map shows that this participant spent significant time outside of his residential neighborhood. This map also illustrates his movement not being isotropic. During this week of observation, this participant traveled mostly in and between north Brooklyn and lower Manhattan.

As an exploratory analysis, we examined GPS feasibility and acceptability by race/ethnicity (i.e. Black, Hispanic, and White) for the different GPS feasibility and acceptability metrics and did not find any appreciable differences (results not shown), suggesting that GPS research is feasible and acceptable for YMSM of different racial/ethnic groups.

## Discussion

This is the first GPS study to be conducted among any sample of MSM. In this study, we evaluated the feasibility and acceptability of GPS methods among a sample of YMSM in the New York City-based P18 Cohort Study, as part of a larger sub-study to understand the spatial context of substance use and sexual risk behaviors. We successfully recruited 75 YMSM, where one of the criteria was being willing to wear a GPS device for a week. Overall, we found that it was feasible and acceptable to use GPS devices among our sample of YMSM. For example, the GPS return rate was 100%. Of the participants with any GPS data, 100% had at least one hour of GPS data for one day and 84.4% had at least one hour on 7 or more days. While this met our goal for feasibility, it should be noted that one hour of wear time may not be sufficient to fully quantify activity spaces and a greater amount of GPS data spanning a longer time duration may be required. In addition, while our success rate for one hour of wear time of 7 days was 100%, this fell off when the wear time threshold was increased. Indeed, when participant data was disaggregated by the number of hours participants wore the GPS, we found that participants had a range of viable data.

It is difficult to compare our results with other GPS studies because no other GPS research has been conducted to date in any MSM population. GPS research (including GPS feasibility research) has been conducted in a variety of populations and generally demonstrates that using GPS devices is feasible and acceptable for different population groups, including marginalized populations. In recent a GPS feasibility study among a sample of 120 low-income housing residents in New York City where they wore a GPS device for a week (the study also used QStarz’s BT-Q1000XT GPS Travel Recorder), participants (mean age of 39 years and 55% female) reported high ratings of pre-GPS acceptability and ease of use and low levels of wear-related concerns in addition to few concerns related to safety, loss or appearance, which were maintained after the baseline GPS feasibility data collection. Our results replicate this finding in the YMSM population. The overall GPS return rate in the New York City study of low-income housing residents was 95.6%. Out of the total of 114 participants with GPS data, 112 (98.2%) delivered at least one hour of GPS data for one day and 84 (73.7%) delivered at least one hour on 7 or more days. The qualitative interviews indicated that overall, participants enjoyed wearing the GPS devices, that they were easy to use and charge and that they generally forgot about the GPS device when wearing it daily [23]. Our results are therefore highly comparable to this study.

We recognize that feasibility and acceptability are likely determined by myriad factors. Feasibility and acceptability were likely influenced by our financial incentive, our use of the travel diary, our sending daily reminder texts to charge their GPS device as well as the weeklong time frame of our GPS protocol. In addition, participants’ were allowed to wear the GPS device in their pocket, which may have increased compliance and might explain our finding a lower proportion of people who reported being concerned with how they looked with the GPS at follow-up. We also note that YMSM may be image-focused (as previous research suggests [63]) and participants were worried about it being bulky and noticeable, but after wearing it for a week they realized it was small and out of view. This study provided a cash incentive, and participants seemed to appreciate this method of compensation. The majority of the participant compensation was given after the GPS was returned and the cash incentive may have influenced the 100% return rate in this study. Furthermore, on the consent form we stated “The purpose of this study is to learn more about how global positioning systems (GPS) technology can be used to help us understand what types of neighborhood conditions influence health and health-related behavior of young men”. As such, here and elsewhere the altruistic motives of study participation were emphasized (i.e., producing useful information to reduce HIV and improve neighborhood conditions for MSM through effective policies), which may have also increased participation rate. Over the course of the cohort study (i.e., past 6 years), we have built a strong relationship with our participants. This relationship has allowed a rapport to be developed, which may have increased trust and therefore feasibility and acceptability of these geospatial methods. The participants’ extensive experience with research, combined with appointment reminders, probably increased compliance as well, but we are not able to quantify by what degree.

### Future Research

Future research utilizing GPS-based methods are warranted and needed among YMSM, including larger samples of YMSM given the health disparities they experience. There should be other GPS feasibility research among MSM samples in different populations (e.g. homeless MSM), different ages (e.g. older MSM populations) and across geographies including rural populations, for example, in the southern United States (known as the Deep South)–where the prevalence of HIV is particularly high [64, 65]. Because our findings highlight the importance of improving GPS protocols, future studies should find ways to increase adherence to GPS charging and wearing protocols. Different and more advanced GPS protocols could be used. For example, future GPS protocols can have longer assessment periods and assessments over time among the same participants, which can help researchers to understand the extent of spatial overlap in people’s spatial patterns over time [66]. This has rarely been done to date and may vary, in part, due to seasonal variation. Indeed, people’s spatial mobility might vary by meteorological seasons (spring, summer, fall, and winter) [67]. Future research could also test the feasibility and acceptability of using a dedicated GPS device (which was used in this study) as compared to other GPS devices (e.g., a GPS-enabled smartphone). Among MSM, there has been some smartphone-based research on geosocial networking smartphone applications like Grindr [68–72], which could be integrated into GPS research on spatial mobility and spatial polygamy. It is also important for future research to examine how characteristics of activity space neighborhoods (measured by GPS devices) can influence health among MSM including substance use and sexual risk behaviors, while accounting for selective daily mobility bias [19, 24]. For example, activity space neighborhood-level lesbian, gay, bisexual and transgender (LGBT) hate crimes may be associated with poor health among MSM [73–75]. To our best knowledge, no research has been conducted examining relationships between activity space neighborhoods and health behavior (e.g. drug use and sexual risk behaviors) in MSM. We encourage researchers to examine a variety of other neighborhood characteristics including neighborhood-level HIV prevalence in one’s activity space: we recently found that incident infections were marginally higher among those residing in neighborhoods with higher rates of HIV prevalence [76]. Because we [76] and others [17] have found stark racial disparities in HIV (e.g. Black YMSM have higher prevalence and incidence of HIV), it could be useful to examine these activity space neighborhood effects by race/ethnicity and importantly results from this study show that GPS feasibility and acceptability did not vary by race/ethnicity (e.g. GPS devices may be used in different MSM populations including Black YMSM). In this future research, it is important to understand mobility patterns and examine “deliberate” neighborhoods (e.g. those that individuals intentionally travel to regularly) versus “incidental” neighborhoods (e.g. those that individuals may go to for a singular occasion or irregularly)—which in part could be done using qualitative methodologies or by extending the assessment period of the protocol, as suggested earlier. In addition to advancing the literature on health and place, this future research will minimize spatial misclassification [10] as well as overcome the residential trap [11, 12]. Furthermore, the proposed future research will inform specific neighborhood-level policy interventions. For example, increasing community efforts to combat LGBT hate crime neighborhood rates through increased local police attention in high-crime locations may be an HIV prevention intervention (if this association exists). Second, given that we will know the travel patterns of MSM, we will be able to identify optimal geographic locations for drug use and HIV testing/prevention interventions, which currently have limitations. Finally, this research will facilitate the identification of geographic locations suitable for recruiting MSM in research studies (an improved method of venue-based sampling).

### Limitations

In spite of this study’s substantial strengths, it also has limitations. In part, we used survey methods to examine the success of GPS feasibility and acceptability. Consequently, participants may have succumbed to social desirability reporting, i.e. the tendency of survey respondents to answer questions in a manner that will be viewed favorably by others. In addition, it is possible that participants could have changed their spatial patterns due to them being GPS tracked, which would lead to potential reactivity bias. However, these issues may be minimal in this study. To illustrate, we asked the question, “Did using the GPS device cause you to alter your behavior?” in the post GPS survey and almost 99% of the sample said “no”. We, therefore, do not believe that the GPS devices altered most participants’ spatial behavior. This study was based on a convenience sample in an existing cohort, which may have consisted of individuals who were more motivated to be in the study and to complete the GPS protocol. By using an existing cohort indeed, there is also selection bias. This could have increased the likelihood of success of the GPS protocol. In addition, while 75 participants is a relatively small sample size for general population health research, given that many recent GPS studies have fewer than 100 participants, our sample size is similar to the sample sizes of most GPS-based research to date. Furthermore, this study was conducted among a relatively small sample of YMSM in New York City and included very few Asian and Multiracial/Other MSM. Additionally, our study was conducted among a sample of predominantly racial/ethnic minority HIV-negative YMSM in New York City. Although relative homogeneity of participants was desired for this feasibility pilot study, these findings, therefore, might only be generalizable to similar MSM populations in urban areas similar to New York City, such as racial/ethnic minority HIV-negative YMSM in urban settings. Because this study was conducted in New York City, a large metropolitan location, issues with large buildings and potential GPS errors due to multipath reflectance are possible [77]. In addition, individuals in New York City often travel via the subway system and while they are underground GPS receivers are unable to obtain signals from GPS satellites, which may lead to additional GPS data loss. Although GPS signals may be lost periodically in the context of a large metropolitan areas, these data are still valuable in determining general activity space and in examining how participants travel throughout different neighborhoods.

### Conclusion

Results from this pilot study demonstrate that utilizing GPS methods among YMSM is feasible and acceptable. GPS devices may be used in spatial epidemiology research in YMSM populations to understand place-based determinants of health such as substance use and sexual risk behaviors.
